# M. Retsius on the Structure of the Teeth

**Published:** 1842-11-26

**Authors:** 


*October* 17, 1842.

*M. Retsius on the Structure of the Teeth.*—According to the author, the teeth of man and the mammalia are composed of three substances—viz., enamel, ivory and cortical substance; these are also found in the teeth of several amphibious animals and fishes.

The ivory contains tubes and cells which communicate with each other, and are identically the same as the small canals and cells which constitute so important a part in the organization of bones. The ivory tubes open towards the cavity of the pulp, in a radiated manner; several of them are, however, parallel, and they send out ramuscules on all sides, extremely fine, forming numerous reticular anastomoses, and terminating in the cells. The thickness of the principal tubes varies from 1-400th to 1-1000th part of a line. As the tubes subdivide, this thickness diminishes, and the minute ramifications are much thinner. The cells and minute ramifications of the tubes finally disappear, but it is probable that those which appear under the microscope, form only a small portion of what really exists in bone.

The ivory is deposited in layers round the surface of the pulp, the external layer being the one first formed, and so on. While this deposition is taking place, the external cells and the peripheral extremities of the tubes are formed; and as the successive layers of ivory are deposited, the tubes become continuous from one layer to another, in such manner that the successive segments of tubes form at last one uninterrupted canal. It would seem that the numerous parallel undulations of the tubes are occasioned during their passage from one layer to another. To explain this, we must admit a periodical movement in the pulp, by which the nascent tubes are at one time carried towards the end of the crown, and at another towards the extremity of the root of the tooth. The finest and most numerous undulations depend on periodical movements of short duration; the more extensive ones are produced by changes in the situation of the pulp, which occur at much longer intervals. The mode of formation of the ivory is analogous to that of bone, and the similarity is greater than might at first sight be imagined ; there is, however, this difference, that in the ivory of a tooth it is the external layer (*couche extreme*) which is first formed, while in bone the same layer is formed last around each medullary filament.

The organisation of the enamel is much more simple; it contains neither blood-vessels nor osseous tubes, but somewhat resembles in structure the crystalline lens. The enamel is probably nourished by imbibition of an organic fluid carried by the ivory tubes, and transmitted through a thin membrane, which probably surrounds the different cells. In some animals the formation of enamel is not confined to the dental follicle, but continues during the whole period of life, through the medium of a small annular apparatus, which envelops the root of the tooth.

The corticle substance exists in the teeth of most mammiferous animals, and even of amphibious animals and fishes. It is peculiarly rich in cells and osseous tubes, which are generally very irregular. in some animals (the elephant, horse, ox) it is formed principally inside the dental follicle, but in others it is formed, during the whole period of life, by the membrane which envelops that portion of the tooth enclosed in the alveolar process. The ivory, as has been said, is deposited in layers from within outwards, the most external being the first formed ; the corticle substance, on the contrary, is formed in an opposite direction, the external layer being the one last deposited. *Prov. Med. Jour.*, Oct. 29, 1842.

